# Living Well With Cognitive Impairment: Social Connection, Well-Being, and Cognition in Very Old Age

**DOI:** 10.1093/geroni/igaf122.2051

**Published:** 2025-12-31

**Authors:** Selina Vogel, Susanne Zank

**Affiliations:** University of Cologne, Cologne, Nordrhein-Westfalen, Germany; University of Cologne, Cologne, Nordrhein-Westfalen, Germany

## Abstract

Research emphasizes that social relationships protect against cognitive impairment. However, their role for individuals already experiencing cognitive impairment is less understood. For them, social connections may be crucial to maintain well-being. Therefore, the present study examined the associations between cognitive impairment, social relationships, and psychological well-being in very old adults (80+ years), a high-risk group for cognitive decline. We analyzed data from proxy- and target-person interviews of 1.561 participants recruited for the Study on Quality of Life and Well-Being in North-Rhine Westphalia (NRW80+). Participants were classified as having no cognitive impairment (66.80%), mild cognitive impairment (16.00%), or major cognitive impairment (17.20%). We compared the groups on three social factors (close network, social activities, and loneliness) and two well-being measures (depressive symptoms and positive affect). Moreover, we investigated whether cognitive impairment moderated the associations between social factors and well-being. As anticipated, cognitive impairment was associated with lower social outcomes and reduced well-being. Additionally, social factors were associated with higher well-being, and these associations were partly moderated by cognitive status. Notably, the association between social engagement and depressive symptoms was more pronounced for individuals with major cognitive impairment compared to the other groups. The findings underscore the significance of social relationships for psychological well-being, especially in the presence of major cognitive impairment. They also demonstrate the general risk of reduced social and mental health faced by individuals with cognitive impairment. Further research is needed, as the topic is increasingly relevant due to the growing rates of cognitive impairment in our aging society.

